# Supplementary material for: Across two continents: the genomic basis of environmental adaptation in house mice (Mus musculus domesticus) from the Americas
Source: bioRxiv. 2023 Nov 2:2023.10.30.564674. Preprint. [Version 1] doi: 10.1101/2023.10.30.564674 (PMC10634997; doi:10.1101/2023.10.30.564674)
Supplement: Supplement 1 [file NIHPP2023.10.30.564674v1-supplement-1.pdf]

bioRxiv preprint doi: https://doi.org/10.1101/2023.10.30.564674; this version posted November 2, 2023. The copyright holder for this preprint (which was not certified by peer review) is the author/funder, who has granted bioRxiv a license to display the preprint in perpetuity. It is made available under aCC-BY 4.0 International license.

Supporting information

Alternative language abstract

Resumen (Spanish)

Resumo (Portuguese)

Alternative language author summary

Resumen del autor (Spanish)

Resumo do autor (Portuguese)

Supplementary Tables

Supplementary Table 1. List of 86 wild-caught house mice individuals (*Mus musculus domesticus*) collected in Mexico (N=10), Brazil (N=60) and Argentina (N=26). Table contains: Collector's number, SRR ID, Museum of Vertebrate Zoology catalog number, exact collecting locality, latitude, longitude, sex, reproductive data, measurements of length and body weight, and data of exome sequencing (number of reads, length of reads and coverage)

Supplementary Table 2. Sample information for the European samples of *Mus musculus musculus, Mus musculus domesticus, Mus musculus castaneus,* and *Mus spretus* from Harr et al. (2016; Doi: 10.1038/sdata.2016.75) included in our analyses.

Supplementary Table 3. Pairwise differentiation (Fst) across the three transects: South America, East and West of North America.

bioRxiv preprint doi: https://doi.org/10.1101/2023.10.30.564674; this version posted November 2, 2023. The copyright holder for this preprint (which was not certified by peer review) is the author/funder, who has granted bioRxiv a license to display the preprint in perpetuity. It is made available under aCC-BY 4.0 International license.

Supplementary Table 4. Values for bioclimatic environmental variables for sampled populations of *M. musculus domesticus* across the Americas.

Supplementary Table 5. Loadings of bioclimatic variables for the first five principal components for the analysis of climate data for all included populations in the Americas.

Supplementary Table 6. Results of Latent Factor Mixed Model (LFMM) analysis for the each of three variables (latitude, MAT, PDM) in South America populations as well as information about top candidates and shared candidates, and the allele frequencies.

Supplementary Table 7. The distribution of candidate SNPs identified in LFMM analyses of South American populations and all SNPs included in the analyses across predicted functional consequence category.

Supplementary Table 8. The results of enrichment analysis for candidates identified in LFMM analysis of South American populations.

Supplementary Table 9. Classification and annotation of SNPs identified as candidates in LFMM analyses of North American populations with LAT, MAT, and PDM.

Supplementary Table 10. Proportion of genes shared between and across all transects for each variable.

bioRxiv preprint doi: https://doi.org/10.1101/2023.10.30.564674; this version posted November 2, 2023. The copyright holder for this preprint (which was not certified by peer review) is the author/funder, who has granted bioRxiv a license to display the preprint in perpetuity. It is made available under aCC-BY 4.0 International license.

Supplementary Table 11. Pairwise permutation test results for overlap among candidate genes for each environmental variable identified across the three transects: South America (SA), Eastern of North America (ENA), and Western of North America (WNA), using a *p-value* £ 0.05 and 10,000 permutations. The number of genes shared for each variable are described in Figure 4.

Supplementary Table 12. Functional information for candidate genes shared across the three transects for each environmental variable.

Supplementary Table 13. Gene annotations and functional information for candidate SNPs identified via GEMMA for body weight.

Supplementary Figure 1. a) Climatic variation across the sampled localities in South America (SA), East (ENA) and West (WNA) of North America using PCA with 19 bioclimatic variables from the WorldClim database. The first component is mainly associated with variation variables relating to temperature (e.g., mean annual temperature, MAT), and the second principal component was mainly associated with precipitation of the driest month (PDM) and precipitation of the driest quarter. **b)** Latitude and Bio1- MAT are significantly correlated across the sampled localities (ENA, WNA, SA). There is no evidence of correlation between **c)** latitude and Bio14-PDM and **d)** MAT and Bio14-PDM across the sampled localities (ENA, WNA, SA).

bioRxiv preprint doi: https://doi.org/10.1101/2023.10.30.564674; this version posted November 2, 2023. The copyright holder for this preprint (which was not certified by peer review) is the author/funder, who has granted bioRxiv a license to display the preprint in perpetuity. It is made available under aCC-BY 4.0 International license.

Supplementary Figure 2. Heatmap of pairwise relatedness coefficients between individuals from the same population using the relatedness estimator R_{AB}, described by Hedrick and Lacy (2014). Individuals that were removed because they were close relatives to another sampled mouse (with a pairwise relatedness value greater than 0.25) are shown in bold.

Supplementary Figure 3. Body weights of adult mice across Eastern of North America (ENA), Western of North America (WNA), and South America (SA) included in the GEMMA analysis.

Supplementary Figure 4. Structure plot showing population genetic clusters using K=2:7 across North and South America.

Supplementary Material. LFMM output, zscores, p-value correction, and allele frequencies for South America, Eastern and Western of North America transects.

Supporting Information.

Alternative language abstract (Spanish and Portuguese)

Alternative language author summary (Spanish and Portuguese)
